# Local constructions of gender-based violence amongst IDPs in northern Uganda: analysis of archival data collected using a gender- and age-segmented participatory ranking methodology

**DOI:** 10.1186/s13031-018-0140-6

**Published:** 2018-02-07

**Authors:** Alastair Ager, Carolyn Bancroft, Elizabeth Berger, Lindsay Stark

**Affiliations:** 1 Mailman School of Public Health, Columbia University, New York, USA; 2 NIHR Research Unit of Health in Situations of Fragility, Institute for Global Health and Development, Queen Margaret University, Edinburgh, UK

**Keywords:** Uganda, Gender-based violence, Marital rape, Rape, Intimate partner violence, Participant ranking methodology (PRM), Internally displaced person (IDP), Social norms

## Abstract

**Background:**

Gender-based violence (GBV) is a significant problem in conflict-affected settings. Understanding local constructions of such violence is crucial to developing preventive and responsive interventions to address this issue.

**Methods:**

This study reports on a secondary analysis of archived data collected as part of formative qualitative work – using a group participatory ranking methodology (PRM) – informing research on the prevalence of GBV amongst IDPs in northern Uganda in 2006. Sixty-four PRM group discussions were held with women, with men, with girls (aged 14 to 18 years), and with boys (aged 14 to 18 years) selected on a randomized basis across four internally displaced persons (IDP) camps in Lira District. Discussions elicited problems facing women in the camps, and – through structured participatory methods - consensus ranking of their importance and narrative accounts explaining these judgments.

**Results:**

Amongst forms of GBV faced by women, rape was ranked as the greatest concern amongst participants (with a mean problem rank of 3.4), followed by marital rape (mean problem rank of 4.5) and intimate partner violence (mean problem rank of 4.9). Girls ranked all forms of GBV as higher priority concerns than other participants. Discussions indicated that these forms of GBV were generally considered normalized within the camp. Gender roles and power, economic deprivation, and physical and social characteristics of the camp setting emerged as key explanatory factors in accounts of GBV prevalence, although these played out in different ways with respect to differing forms of violence.

**Conclusions:**

All groups acknowledged GBV to represent a significant threat - among other major concerns such as transportation, water, shelter, food and security – for women residing in the camps. Given evidence of the significantly higher risk in the camp of intimate partner violence and marital rape, the relative prominence of the issue of rape in all rankings suggests normalization of violence within the home. Programs targeting reduction in GBV need to address community-identified root causes such as economic deprivation and social norms related to gender roles. More generally, PRM appears to offer an efficient means of identifying local constructions of prevailing challenges in a manner that can inform programming.

## Background

Gender-based violence (GBV) has, over the last 20 years, emerged as a major concern within populations in conflict-affected settings [1]. GBV has potentially severe implications for wellbeing, causing physical pain and disability, adverse mental health outcomes and increased risk of Sexually Transmitted Infections (STIs) and unintended pregnancies. Programming by non-governmental agencies to address the needs of survivors – and to prevent perpetration of such violence – is now established within the mainstream of humanitarian response, and coordinated with respect to established inter-agency guidelines [2].

Quantification of the scope and magnitude of GBV is especially challenging in humanitarian settings, with an even greater likelihood of fear and secrecy than in other settings leading to suppression of reporting [3]. Nonetheless, an increasing number of studies have confirmed the high prevalence of GBV in such contexts, though estimates vary due to different methodologies and measures used in surveys in complex emergencies [1, 4–9]. An assessment of prevalence of GBV in internally displaced persons (IDP) camps in Northern Uganda found that 50% of women reported experiencing some form of violence in the previous year, 40% reported forced sex with an intimate partner and 5% reported having been raped by someone outside of their household [10].

While these studies have confirmed the importance of GBV as an issue, they provide an inadequate evidence-base to inform programming. It is important to identify risk and protective factors for such violence, and the means by which this information may be used to plan more effective prevention and response programs. For example, a study in Kakuma refugee camp in Kenya featured 18 refugee focus group discussions exploring the nature and consequences of intimate partner violence (IPV) in the camp and responses to IPV [11]. The study provided insights not only into how people interpreted the issue of IPV but also what were considered to be more effective response mechanisms such as traditional, familial or community-based responses rather than agency-led, formal interventions. Wirtz et al. used focus group discussions to investigate barriers to reporting and service-seeking efforts amongst refugees in Ethiopia [12]. Discussions revealed rich information about types, perpetrators, causes and contexts of GBV, which were used to develop a screening measure for use in similar contexts. A systematic review of predictors of interpersonal violence in humanitarian settings identified four modifiable risk factors for violence against women and children: substance abuse, economic status, mental health and social support (manuscript currently under review). Currently, examples of published studies investigating local understandings of the factors influencing GBV are uncommon. There is a need for more examples of such work, especially those which can not only shape local response and prevention strategies but also wider understanding of the dynamics of GBV in conflict-affected and other humanitarian contexts.

This paper reports on such a study, drawing upon archived data collected in the context of developing GBV programs for IDP populations in northern Uganda in 2006. In northern Uganda, the early years of the millennium saw the population seeking to recover from nearly two decades of civil war. Insurgency by the Lord’s Resistance Army, led by Joseph Kony, and Government of Uganda military operations resulted in the displacement of over 1.8 million people, disrupting community structures and previously defined social roles, destabilizing families and interrupting economic activity [13]. In 2006, many were still residing in over 150 IDP camps for internally displaced persons (IDPs) that had been established during the conflict in the northern part of the country. Access to food, clean water and basic services such as medical care were often inadequate in these settlements [14]. The context and conditions of camps prompted consideration of GBV programming as a key area of humanitarian response across camps throughout the region. In Lira District, ChildFund^1^ led such programming response, and commissioned an assessment of prevalence of GBV to assist in program development and evaluation. This survey – the findings of which were noted above [10] – established one-in-twenty women to have experienced rape by a non-domestic partner and one-in-two to have experienced intimate partner violence in the previous twelve months.

This study reports on a secondary analysis of archived data which had been collected through formative participatory research in support of this prevalence survey and subsequent program development. This participatory work had sought to elicit local construction of GBV experienced by women in the camp, explicitly seeking the understandings not only of women themselves but also of men and, acknowledging the potential of perceived norms in shaping future behaviour, of girls and boys. The archived data presented a window into the social experience of GBV in camps in northern Uganda in the period after the formal end to civil hostilities - but before widespread resettlement from the camps - which stood to inform current debates within the literature identified above. It also provides an opportunity to examine the potential utility of participatory ranking methods as a means of rapid appraisal of key local understandings and agendas in the context of a humanitarian setting.

## Methods

### Setting

Data collection was conducted in four camps for IDPs in Lira District, Northern Uganda. The camps - Aromo, Ayami, Walela and Okwang - were selected on the basis of their not having been the focus of interventions addressing GBV by ChildFund or any other agency, but being the proposed or potential focus of such intervention subsequent to the fieldwork described. Table 1 gives details of the populations of the four camps.

**Table 1:** Demographic Profiles of the Four Studied Camps

Camp	Aromo	Ayami	Walela	Okwang
Estimated Total Population	24,828	9068	7200	6348
Males	12,006	4464	3269	2539
Females	12,822	4604	3931	3809
Households	5407	2022	1571	1269

### Facilitators

Four facilitators (two women, two men) were trained to facilitate group discussions using a participatory ranking methodology (PRM) [15]. They were selected as having experience of working in the field of gender-based violence programs and having *Luo* (the predominant local language spoken in the District) as their first language. Training considered local language usage and definitions with respect to relevant terms. Three forms of gender-based violence were particular foci for the study: intimate partner violence (defined as physical beating by an intimate partner), marital rape (defined as forced sex with an intimate partner through the use of physical violence, its threat, or other coercion) and rape (defined as sexual intercourse, or attempted sexual intercourse, without consent by someone other than an intimate partner). Facilitators worked in same-gender pairs (one female pair, one male pair) allowing their gender to be congruent with that of participants. For each group discussion, one facilitator took the role of discussion lead, while the other took the role of note-taker.

### Participants

In each of the four camps a series of PRM group discussions were held. In each camp groups were held with women, with men, with girls (aged 14 to 18 years), and with boys (aged 14 to 18 years). While criterion sampling was used to ensure groups were composed of camp residents of the appropriate age and gender, the first participant of adult groups was identified through random sampling in order to convene a more representative range of the camp population, rather than those in contact with services. This ‘convener’ was then invited to assemble 8–10 other participants meeting the age and gender criteria of that group. Blocks were selected using a random number sequence. Within each block a sampling interval, *m*, was calculated by dividing the estimated number of households by 30. The initial location where a group ‘convener’ was sought was determined by using a random number sequence to select a number, *s*, within this sampling interval. Interviewers approached the selected household (counting *s* households from the boundary of the block) and sought – according to requirement for a women’s, men’s, girl’s or boy’s group – consent from a member of the household of the appropriate age and gender to identify between nine and eleven others (of the same age and gender) to form a group for discussion. If no appropriate ‘convener’ could be recruited at this household (which was reported in less than 10% of sampled households), interviewers continued past a further *m* households. This process was repeated until the targeted number of groups was achieved in each camp. Half of all boys and girls PRM groups were constructed by quota sampling from school registers. The remainder were convened using an analogous approach of household sampling to that adopted with adult groups (which ensured out-of-school children were recruited into discussions). The total number of PRM groups completed in each camp is given in Table 2.

**Table 2:** Number of Focus Group Discussions Across the Four Studied Camps

Camp	Aromo	Ayami	Walela	Okwang
Women	4	4	4	4
Men	2	6	4	4
Girls	4	4	4	4
Boys	3	5	4	4

### Protocol

The selected ‘convener’ invited other participants for what was described as an activity discussing ‘the issues facing women and girls in the camp’. Focus groups were convened in areas away from the main thoroughfares of the block (or school). On coming together it was explained to participants by the facilitator that this was an activity that would last for approximately half an hour. Participation had no bearing on receipt of services, and participants could discontinue the activity at any time without consequence. Participants were then verbally asked if they consented to continue. Given the nature of group interviews where the focus was not on reporting of personal experiences but of perceptions of wider circumstances facing others, children aged 14 and above were deemed capable of providing consent for participation in the same way as adults by local child protection advisers. Facilitators explained at the beginning of each session that individuals should speak freely, and who said what would not be recorded. At the end of each session, facilitators thanked the group for their participation, reminded participants that names had not been recorded, and provided information on where referral services could be accessed. The study was reviewed and approved through the Columbia University Medical Center IRB. In addition, the work was undertaken with clearance by the Ugandan Office of the President for informing and evaluating planned humanitarian interventions. Before the commencement of data collection, approvals were also secured from block leaders and school head teachers, who assisted in implementing a sampling strategy that ensured the equi-probability of selection of PRM group conveners from across the camp.

Consenting participants were then asked by the facilitator to identify ‘some of the major issues facing women and girls in the camp’. For each issue that was suggested, the facilitator identified – with the assistance of participants – a physical object to represent that issue (e.g. a stone, a household object, an image drawn on paper) after the fashion described by Ager et al. [15]. A ‘pile’ of such objects was assembled, the facilitator encouraging participants to consider if new suggestions warranted a new object or if existing objects already covered the suggestion adequately. The process continued until a maximum of ten issues (and thus objects) had been identified. If, after six objects had been identified, the issues of intimate partner violence, marital rape and rape had not been mentioned, the facilitator prompted participants to consider whether these issues warranted inclusion in the ‘pile’. The note-taker documented verbatim comments made by participants justifying the inclusion or exclusion of all issues.

Once the ‘pile’ was assembled, the facilitator drew a line on the ground, and asked participants to consider the relative importance of these issues in the lives of women and girls in the camp. Modeling selection of an object and placing it at a point on the line representing ‘very important’ or ‘less important’, the facilitator encouraged participants to select an object and place it at an appropriate point on the line. Participants were asked to verbally justify their placements. Readjustment of the placement of objects by participants was encouraged until the line of objects was taken to represent the consensual view of participants regarding the appropriate relative prioritization of issues. Throughout the process of placement and readjustment of objects, the note-taker recorded the justifications given for proposed positioning of the various issues, which not only facilitated ranking but also negotiation of the drivers and consequences of reported problems. Participatory activities thus elicited both quantitative problem rankings and elaborated qualitative narrative regarding norms, risk factors and impacts of the highlighted forms of GBV.

### Analysis

Analysis of this archival data consequently utilized both quantitative and qualitative approaches. Comparison of rankings within and across groups used non-parametric tests available through SPSS [16]. Our analysis was conducted with the assumption that the underlying distribution was different across groups so we report means rather than medians. An inductive thematic analysis of narrative followed entry of all text into NVivo, open coding of the material, with axial codes then developed to identify major explanatory themes.

## Results

### Quantitative findings

We used use Friedman tests to compare means across groups on the basis that sampling processes drew together groups of similar individuals from the camp populations. Overall the mean problem ranking of rape was 3.4 (1 representing greatest importance) amongst major issues faced by women and girls. This was a significantly higher ranking than for both marital rape and intimate partner violence, which secured a mean problem rank of 4.5 and 4.9 respectively (Freidman, *n* = 60, χ^2^ = 20.1, *p* < .0005].

Table 3 shows the variation in mean ranking of forms of GBV by gender and age; rankings differed significantly for rape (Freidman, χ^2^ = 15.1, *p* = 0.002), marital rape (χ^2^ = 8.5, *p* = .036) and intimate partner violence (*χ*^2^ = 11.1, *p* = 0.011). Post hoc analysis with the Wilcoxon signed-rank test with Bonferroni correction indicated that girls identified rape as a significantly more pressing threat than any other group, with a mean rank of 1.6 compared to women, men and boys rankings of 4.2 (Z = − 2.8, *p* = 0.006), 4.4 (Z = − 3.2, *p* = 0.001) and 3.2 (Z = − 2.4, *p* = 0.015) respectively. Further, girls ranked all forms of GBV significantly higher than boys (Z = − 2.4, p = 0.015; Z = − 2.9, *p* = 0.004; and Z = − 3, *p* = 0.003, for rape, marital rape and intimate partner violence respectively). No significant difference in scores was found between women and men, nor between boys and men.

**Table 3:** Mean Ranking of GBV Issues by Gender and Age of Participants

	IPV Rank	MR Rank	Rape Rank
Overall Mean	4.9	4.5	3.4
Women	5.0	4.2	4.2
Men	5.4	4.9	4.4
Girls	3.5	3.2	1.6
Boys	5.6	5.4	3.3

IPV signifies intimate partner violence, MR signifies marital rape or rape by a domestic partner, and Rape signifies rape other than by a domestic partner

### Qualitative findings

Focus group participants consistently identified the pervasiveness of three contextual factors in facilitating gender-based violence: gender roles and power, economic deprivation, and physical and social characteristics of the camp setting. These factors, although interacting, appeared to play out in somewhat different ways with differing forms of violence. Gender roles and power, for example, were particularly linked in people’s understanding with marital rape and intimate partner violence. Aspects of the camp setting (location of water sources, firewood, bars, and markets) were widely understood as contributing to risk of rape. Economic deprivation was seen as contributing to all forms of GBV. These factors – and narratives illustrating them - are elaborated below. Additional issues related to living in IDP camps such as water, transportation, schooling and access to health care were raised in discussions; however, this manuscript focuses on the consideration of GBV issues.

### Gender roles and power

Male and female respondents alike clearly and uniformly articulated the expected responsibilities and roles of women within the household and in relation to men.

*Traditionally, a woman was taken from a man’s rib that means a woman has no rights, she should be submissive to her husband but some women when you marry them, they want to take the whole responsibility in the family. -* Male Participant, Ayami Camp

Group participants emphasized communally accepted roles of women and girls related to the domestic sphere.

*Parents beat girls when they go to school, saying that…girls work is…to [be in] the garden and [to] cook food –* Girl Participant, Okwang Camp

Central to this communal construction of gender is the role of women as property of their husbands or fathers, and the understanding of their lack of agency to make personal and sexual decisions. One man in describing power dynamics between husband and wife explained, a “woman [will] pretend to be respectful when she’s still new but when she has taken long with you, she will never listen unless beaten” - Male Participant, Ayami Camp. Gendered power relations between men and women contributed to violence within both bodily and domestic spheres.

In discussions of intimate partner violence and marital rape, the perceived sexual obligation of women toward their husbands was understood to trump personal volition. Feminine sexuality, alluded to as “husband’s food” by an Ayami camp elderwoman, is understood to be regulated by men. One woman explained:

*when [my] husband comes back home be it lunchtime or evening, I make sure I send the children out immediately as he lies impatiently on the bed waiting like an angry lion wanting to eat meat. -* Female Participant, Walela Camp

Women who challenged requests for sex – whether because of menstruation or lack of privacy in front of neighbors or their children – were criticized by male group participants for not fulfilling the needs of their partners, or were accused of adultery. Sexual refusal was cited as a key trigger provoking marital disputes and intimate partner violence.

*a man may demand for sex from a wife but, because of fear of children and lack of proper accommodation, a wife may resist -hence fighting. -* Female Participant, Walela Camp

This perceived male hegemony over women’s sexual behaviour was nonetheless fragile. Marital rape was often discussed in connection to perceived female infidelity within marital relationships. Affairs were assumed by men to make women less likely to satisfy the sexual needs of their primary partners, and therefore seen to excuse the actions of male partners who would invite forced marital sex. One man suggested, “some women go with other men and when their husband wants to fulfill their sexual need, they deny them, hence forcing these men to fight and rape them” - Male Participant, Okwang Camp. Men’s fear of the possibility of their wife committing adulterous acts, real or perceived, was consistently linked with marital violence, forced sex and divorce.

Marital rape, along with other forms of violence against women, was also frequently justified as a result of drunkenness. Social drinking practices and subsequent drunkenness of men were cited as prompting and aggravating violence against women, specifically within the household. “Men are beating women in the camp at times they come when they are drunk and beckon you,” and “they are cruel and harsh [to] you sexually without…shame” - Female Participant, Okwang Camp. One respondent reported that when a husband:

*is drunk, even if the children are there, he tells her that he wants sex, [and] if she begs to resist he beats her up and at times he forces her for sex. -* Female Participant, Okwang Camp

Attacks inflicted by drunken men seem to grant the perpetrator a degree of immunity from full responsibility and assumption of blame. Violence associated with drunkenness was described in ‘normal’ and expected terms.

### Economic deprivation

In all group discussions, the theme of extreme household poverty within the camp communities was emphasized as one of the key underlying factors contributing to the pervasiveness of sexual and physical violence. In several examples, limited financial resources provoked marital arguments over monetary allocation, which led to intimate partner violence. In other examples, economic desperation and the lack of income to procure food and essential personal and household items were suggested to be key forces guiding women and girls towards engaging in transactional sex with men in hopes of attaining financial security for themselves and their families.

*In an attempt to meet the demands of their families, [some girls] give their bodies to men who have money. This money is used to buy a few of the basic needs in the family. -* Female Participant, Ayami Camp

Economic deprivation and the lack of material resources—including but not limited to sufficient food rations, water, clothing and property—were highlighted as factors that came to amplify social and gender based tensions already present within the household and the larger community.

The economic desperation of girls and their families was frequently identified as a central motivational force prompting transactional sexual exchanges with soldiers, police officials, and older men. Young girls are described as being tempted and “deceived with money” (Female Participant, Okwang Camp) from men who are viewed, both symbolically and literally, to grant economic protection in exchange for sexual intimacy. “Poverty here paved ways for many problems for young girls,” reflected one male respondent from Okwang Camp. “Rape is very common especially due to poverty in the camp, defilement being inflicted by some business men” - Male Participant, Walela Camp. Soldiers were described to be effective in their efforts coercing women to engage in sexual interactions based on the promise of financial gain. One male participant from Ayami camp reflected to the group, with such a promise “can you refuse?”

The participation of parents and family members in brokering these informal agreements out of economic despair was frequently noted.

*The problem of poverty is causing domestic violence and rape in such a way that some women sell their young in the names of marriage to people with money. -* Male Participant, Okwang Camp

Refusal to comply often led to cases where the girl would be “beaten seriously by the father.” (Girl Participant, Okwang Camp). Alternatively, parents were reported to be “forcing their daughters into early marriage due to high rate of poverty in the camp” citing that “it is better for these girls to get money than to be taken freely without any pay” (Boy Participant, Okwang Camp). The impact of economic deprivation and resulting norm of early and forced marriage is a particularly salient threat for young girls.

Lack of food and monetary resources was repeatedly cited, as both an etiological and an aggravating factor, in association with marital disputes, intimate partner violence and marital rape. One participant asserted that:

*selling food by the husband for drink causes violence. Sometimes you find that when the food is given by WFP, the woman can decide to take it to her parents so if the husband hears, he comes back and beats the wife seriously. -* Boy Participant, Okwang Camp

In Ayami camp, “a wife was beaten by the husband because she had refused him to sell off the saucepan just for paying the debt he had with the owner of the beer” (Female Participant, Ayami Camp). This struggle over resources as a factor precipitating violence is further highlighted in the comments of one man who explained:

*every women ha[s] been beaten by their husband since there is always struggling for food [and], non-food items where one may decide to sell the household properties. -* Male Participant, Walela Camp

A woman from Walela Camp emphasized the protective and preventative benefits of adequate food security with the statement: “If we have food, we can have strength to fight the rapist[s].” Many accounts elaborated the complex linkages between food and violence. As two women from Ayama Camp reported “If the house is not peaceful even food cannot be eaten” and “I prefer being beaten while my children eat.”

Poverty was also perceived to be related to marital infidelity whereby “many women to resort to adultery in search for food [and] money” (Male Participant, Walela Camp). One man from Okwang Camp commented:

*since our women do not have anything to do which can generate income—[it] has led to high rate of adultery especially with the soldiers. They tend to go to soldiers for the sake of money and other non-food times.*

These transactional exchanges were cited as frequent prompts of marital conflicts; one woman from Okwang Camp cited a case where “a woman was given money by a soldiers who later slept in her house and when the husband came back, he fought with the soldier almost causing death.”

### Threats linked to the social and physical characteristics of camps

Social and physical aspects of the camp setting contributed to increased vulnerability to GBV beyond the issues of gender inequity in power relations and economic deprivation discussed above. Participants consistently articulated the role of soldiers, police officials and wealthier males as key perpetrators of violence inflicted upon young girls.

*Most of young girls are... defiled by soldiers mainly. Soldiers come at night while people are sleeping and enter the room by force. -* Boy Participant, Walela Camp

Elevated by social capital and armed with weapons, soldiers and members of militia groups were described as often forcing women and girls to acquiesce to their material and sexual demands.

*some soldiers come and request our daughters to collect for them drinking water from their house, and as they go in, the men follow them into the house and [they are] raped*. - Female Participant, Walela Camp

Abuse from soldiers, in addition to sex of a transactional nature, added to the vulnerability of women and girls in the camp setting.

Equally, if not more frequently cited however, was the role of husbands, family members and neighbors as perpetrators of violence. This correlates with the epidemiological data collected in the parallel survey study in these camps indicating that the majority of gender-based violence was inflicted within the home and within familial circles, rather than chance attacks by strangers in the external environment [3, 10]. The impact of the war and life within the camp was described as aggravating men’s social behavior. “The men have become mad,” one female respondent from Walela Camp said, “they go drinking, watching videos and come back home late, beat and chase us out of our houses.”

Although reports of domestic and marital abuse were dominant, the prevalence and severity of external attacks should not be minimized. Respondents from all four camps recounted examples of women and girls who were targeted by strangers, emphasizing specific geographical locations as axes of vulnerability. Specifically, unprotected areas on the periphery of the camps such as water sources, roadsides and bush areas where firewood is collected were noted in many incident accounts and were therefore associated with high risk. Water sources and spaces on the outer boundaries of camp confines where firewood is collected were also recognised as high-risk areas. According to one female respondent:

*You can be going for fire wood, and when you meet a soldier or police on your way they tend to scare you with [their] gun and just rape you without you making any alarm or reporting to any authority. -* Girl Participant, Okwang Camp

Another girl participant from Okwang Camp observed that “the soldiers wait for you at the water points and rape you.”

Additionally, locations within the camp where men come to congregate socially, including bars, cinemas, military barracks, and local markets were seen to put women, and specifically young girls, in increased danger of harassment and violent attack. The association between physical space and vulnerability to assault was widely recognized by participants. One Walela man, emphasizing the level of vulnerability, claimed: “there is a beer bar in the camp that whichever female crosses by 7:00 pm is raped.” The recognition and normalization of this geographically–associated risk implicitly places both responsibility and blame upon the victims of violence. One girl from Okwang noted that girls should “stay closely with the parents, [and] should not move alone into lonely places.” Communally identified high-risk locations thus become areas that women and girls should know to avoid, with the implication that attacks are to be expected.

Much of this geographical risk is linked to the experience of recent migration and the social dynamics imposed by the immediate camp environment. Restitution is seen to lie in a return to pre-war conditions, a nostalgia for the calm of earlier village life. “How should we avoid defilement?” posited one girl in Okwang, “People should be taken back to villages. The by-laws should be put in place” (Girl Participant, Okwang Camp). In addition to geographic vulnerability, inequity in community relationships was seen as increased in the camp setting. During discussions, women’s sexual obligations to their husbands or partners were linked to forced sex within the home, often associated with lack of privacy within the camp setting and a sense of shame. One Walela respondent recounted:

*there is a man who always comes back home drunk, pulls the woman even when cooking to first fulfill his demands and every day at lunch time he must [have] sex with the wife shaming both the neighbors and the children. -* Female Participant, Walela Camp

Due to limited household space and privacy, the woman herself is often “ashamed before her children” (Female Participant, Okwang Camp). In other instances, the children are also implicated in the marital abuse, occasionally as additional victims themselves. As one Okwang woman recounts, “one time th[at] very man called the daughter, telling her that he could have [sex with] her together with the mother.” Spatial constraints interact with unequal power dynamics and the perceived endemic nature of violence, contributing to greater abuse within the camp setting.

### Impact of abuse

The above sections have considered the three major sources of vulnerability for GBV identified through participant narratives. These narratives also highlight perceptions of critical impacts of GBV on the community in such areas as health and development, wider social norms and community response.

Respondents closely associated the increased sexual and physical violence inflicted upon women and girls as imposing significant physical and social harm. Collectively, groups from all four communities listed ‘defilement,’ disease, injury, depression and shame with the victims of violence. The effects of abuse were understood to be amplified for young girls, who as targets of violence, were seen to be ‘spoilt’(Male Participant, Walela Camp), ‘tarnished,’ (Boy Participant, Walela Camp) and left without a future. Further, abuse was discussed as a barrier to educational success and development; “rape makes one not to enjoy the fruits of her studies” one girl from Walela suggested. Most directly, gender-based violence was considered a significant risk to physical health. As you respondent stated bluntly, “If a man rapes you, one contract disease and die.” *-* Girl Participant, Walela Camp.

Another impact of GBV was its perceived inter-generational influence. Early exposure to household sexual exchanges within the home was often associated with premature sexual awareness and imitation amongst children:

*Some parents…[have] sex when the children are aware and that thing has really spoilt children... you find them imitating what the parents do with their fellow friends. -* Girl Participant, Okwang Camp

Marital rape in particular was cited as teaching youth “dirty acts,” (Female Participant, Walela Camp) and “bad manners [that] makes them ashamed” (Girl Participant, Walela Camp). “It makes children to begin practicing sex early,” one school girl from Walela Camp noted. A woman from Okwang Camp described a case where “some children were found [hav]ing sex and when they were asked they said that they saw from their neighbor.”

Some respondents placed significant blame on the victims themselves who were perceived as “not morally good” (Female Participant, Okwang Camp) and to be inviting the advances of men and withholding their sexual activity from family members.

*Our girls here in the camp are good during daytime but at night, they [are] begging, barking like dogs together with their fellow young boys along the main road. -* Male Participant, Ayami Camp

In this way, young girls are seen to be active participants in sexual exchanges, “indulging [in] sexual immorality” (Female Participant, Okwang Camp) and the pursuit of men, and deceptive to their elders. As one participant shared, one girl “deceives her mother that she is going to school and yet she sleeps with men” (Girl Participant, Walela Camp).

Several respondents recounted instances where men had boasted to their peers about actions inflicted upon women as symbolic of masculinity and familial control. “When men gather at the drinking places, they talk about the rape or dirty things [that] they did the day before” (Female Participant, Walela Camp). One woman from Walela Camp explained, “marital rape is just rampant, and men feel proud to talk about it at their drinking joints.” Less frequently, gender-based violence was discussed as a normalized “sign of love” between husband and wife (Female Participant, Walela Camp). For whatever function, the normalization of violence against women and girls indicated by our ranking data was consistently reinforced:

*Women in this camp take marital rape as being normal. If your husband wants sex, even when you are menstruating, you just have to give in without refusing—even when children are awake. -* Girl Participant, Okwang Camp

The ubiquity of violence – and the erosion of social mores - was emphasized by some individuals noting that religious affiliation or responsibility was no barrier in the perpetration of such acts:

*We have always beaten and harassed our wives. There is a lot of quarreling between the couple even the servants of God are beating their wives. -* Male Participant, Okwang Camp

*With domestic violence, no one in this camp can escape that. We have come to realize that even pastors are committing this crime seriously, so domestic violence is really a big problem. -* Male Participant, Okwang Camp

Despite general acknowledgement of gender-based violence as a fundamental problem in need of attention within their communities, group respondents emphasized that the vast majority of incidents went unreported and that women were often reluctant to make attacks public. When abuse is made public and reported to the authorities, it is seen to be ignored. Expressing frustration about the lack of response from officials, one woman in Okwang asked rhetorically:

*How should we prevent rape…since it is rampant in our camp and it is being [ignored] by the law keepers? -* Female Participant, Okwang Camp

Participants also emphasized the generalized communal apathy toward signs of distress, with acts of rape often ignored by witnesses. “Even if [women] make an alarm in the night, no one ever comes to their rescue” (Female Participant, Walela Camp).

Although impunity was widely acknowledged, several respondents reported examples which highlighted the potential influence and power of camp leaders and police officers in prevention and response. In one case from Okwang camp:

*A 50 year old woman…raped by a soldier…made an alarm [and] then people surrounded them and the soldier was arrested…The case was brought to the barracks [and] he was seriously beaten but was not made to pay anything. -* Female Participant, Okwang Camp

Arranged marriages between the victim’s families and the perpetrator were frequently cited. In one reported case from Okwang camp:

*A girl was raped by a soldier…because the parents wanted money, they had to accept 50000 and one goat. The case ended there with the girl forced to go with that soldier. -* Girl Participant, Okwang Camp

In other instances, payment required by local leaders to assist in the resolution process was prohibitive for families seeking assistance. “If a man beats them, they go to the block leader to settle their case and the block leader in turn demands for some money which discourages them” (Female Participant, Ayami Camp).

## Discussion and conclusion

As noted previously, the archival data analysed here was initially collected as formative research to inform a prevalence survey of sexual violence in the IDP caps of Lira and an appropriate GBV programming response in these settings [14]. Summary data from PRM group discussions supported the conclusions of the prevalence survey that, while there were risks of sexual violence outside of the home (the prior focus of GBV programming), risks within the home were far greater. This led to a reformulation of ChildFund’s programming strategy to more directly address prevention and reporting of household violence.

The subsequent quantitative and thematic analysis of this archival PRM data has not only confirmed that rape, marital rape and intimate partner violence were perceived as serious issues for women within the IDP camp community from the perspectives of both women and men and both girls and boys – albeit with important variations in ranked salience with respect to other threats. The analysis has identified recurrent themes in explaining vulnerability to GBV: gender roles and power, economic deprivation and physical and social characteristics of the camp setting. Discussions across four camps suggested that violence was widely accepted. These findings are consistent with previous studies in post-conflict settings, which have indicated that both men and women perpetuate gender inequitable norms related to sexual relationships and reproductive health, which may prove to be a barrier to the promotion of safe sexual practices such as equitable decision-making, and the prevention of pregnancy and STIs [17].

This study is not without limitations. First, as an analysis of archival data, the study clearly does not speak to the contemporary context of northern Uganda, where IDP camps have largely closed. Second, facilitators only probed about GBV issues if they were not mentioned and did not probe for other specific issues. However, while this prompting may have influenced participant ranking choices, the ranking of GBV varied significantly across groups, suggesting that this procedure did not lead to widespread inflation of ranking GBV issues. Third, given the sampling and group ranking methodology adopted, the analysis of group differences formally only extends to sampled participants rather than the population from which they were drawn (although the extent to which findings are in line with those of other, population-based studies in camp settings suggests their potential representativeness). In consequence, despite these limitations, we consider the study illuminates important risk factors for - and effects of - GBV from the perspectives of women, men, boys and girls.

Gender-based violence impacts women, girls and communities on a variety of social and physical levels. By tracing the root causes of such violence through PRM, dynamics were identified that have the potential to influence the design of programmatic interventions to address prevention activities, as well as justice mechanisms and community response. The heightened concern of young girls regarding GBV compared with adult women and males of all ages suggests that acculturation around the issues of gendered violence reflects both developmental age and gender. Programs seeking to shift social norms need to be strategic in targeting young boys before acceptance of violence against women and girls is well established.

Further, our findings suggest that a comprehensive response to GBV in IDP camp settings of forced displacement will need to address gender roles and conceptions of masculinity, not only through women’s empowerment programming but also by understanding the social impact of displacement on men [18]. Economic strengthening programming in IDP camps may provide greater equity in access to resources (including food and NFIs) and increased economic support interventions. Evidence is amassing that suggests that thoughtful livelihoods initiatives can mitigate GBV [19, 20]. Spatial and social threats within the camp due to proximity of military forces and distance from resources such as firewood and water need also to be considered in design, layout and camp oversight. Additionally, community justice mechanisms in IDP camps should be strengthened to encourage reporting and response. These largely preventive measures need to be complemented by culturally appropriate psychosocial support of survivors [8]. This analysis points to drivers of GBV in humanitarian situations– and mechanisms for their disruption – which are of broad potential relevance across analogous cultural and contextual settings.

## Abbreviations

GBV: Gender based violence
IDP: Internally displaced persons
IPV: Intimate partner violence
IPV: Intimate partner violence
MR: Marital rape
NFI: Non-food items
PRM: Participant ranking methodology
STIs: Sexually transmitted infections

## Footnotes

1 At the time operating as Christian Children’s Fund (CCF)
